# Empowering Support for Family Members of Patients With Traumatic Brain Injury During the Acute Care: Insights From Family Members and Nurses

**DOI:** 10.1111/jan.16424

**Published:** 2024-09-05

**Authors:** Julia Lindlöf, Hannele Turunen, Kirsi Coco, Justiina Huhtakangas, Sofie Verhaeghe, Tarja Välimäki

**Affiliations:** ^1^ Department of Nursing Science University of Eastern Finland Kuopio Finland; ^2^ Kuopio University Hospital Kuopio Finland; ^3^ Helsinki University Hospital and Helsinki University Helsinki Finland; ^4^ Faculty of Medicine and Health Sciences, Department of Public Health and Primary Care University Centre for Nursing and Midwifery, University of Gent Ghent Belgium; ^5^ Department of Nursing VIVES University College Roeselare Belgium; ^6^ Faculty of Medicine and Life Science University Hasselt Hasselt Belgium

**Keywords:** acute care, empowering support, family members, neurological care, neurosurgical care, nurses, patients with traumatic brain injury, practice development, qualitative approaches

## Abstract

**Aim:**

To investigate the perceptions of family members (FMs) of patients with traumatic brain injury (TBI) and nurses on empowering support and its implementation during the acute phase within Finnish neurosurgical and neurological care in hospital settings, focusing on identifying similarities and differences in their viewpoints.

**Design:**

Participatory qualitative descriptive study.

**Methods:**

Data were collected from seven FMs and 11 nurses using the World Café method in November 2019. An abductive approach was employed for data analysis, combining deductive interpretation within the conceptual framework of empowering support and inductive content analysis.

**Results:**

Four main themes were identified: (1) FMs' diverse information and guidance needs of TBI, treatment and its impact on family life, (2) support based on empowering FMs in participation, competence and decision‐making, (3) empowering FMs through collaborative nursing practices and interprofessional support, and (4) internal and external hospital support enhancing and promoting the empowerment of FMs.

**Conclusion:**

The perceptions of FMs and nurses regarding empowering support were largely consistent, yet diverged in its implementation in nursing practice. Nurses play a crucial role in fostering the empowerment of FMs; however, further research is needed to explore the impact of organisational and community factors on the implementation of empowering support.

**Impact:**

Our study contributes to advancing nursing practices by underscoring the necessity for a paradigm shift towards a family‐centred approach. Furthermore, it emphasises the urgency for standardising nursing practices to ensure equitable access to empowering support for FMs, applicable across various care settings for patients with TBI.

**Public Contribution:**

This review is part of a larger research project in which FMs of patients with TBI and nurses were involved in designing the project.

**Reporting Method:**

This study was reported using the Consolidated Criteria for Reporting Qualitative Checklist for qualitative studies.

**Summary**

**Why Is This Research or Review Needed?**

- There is limited understanding of how empowering support for family members is perceived and implemented by both family members and nurses in acute care settings for patients with traumatic brain injury.
- This study addresses the gap by exploring these perceptions, aiming to improve nursing practices and patient outcomes through a family‐centred approach.

**What Are the Key Findings?**

- Four main themes emerged: diverse information needs, support through participation, empowerment via collaborative practices and enhancement through internal and external support.
- Although family members and nurses agreed on the importance of empowering support, their perspectives differed on its implementation in practice.
- The findings underscore the critical role of nurses in facilitating family member empowerment through collaborative and interprofessional practices.

**How Should the Findings Be Used to Influence Policy/Practice/Research/Education?**

- Nursing practices should transfer towards a family‐centred approach, emphasising standardised practices to ensure equitable support for family members.
- Further research should explore organisational and community factors that affect the implementation of empowering support to improve clinical outcomes.
- Educational programmes for healthcare providers should integrate these findings to enhance training and practice related to empowering family members.

**Impact Statement**

- Enhances understanding of the importance of empowering support for family members in neurosurgical and neurological acute care for patients with traumatic brain injury, fostering family‐centred care.
- Highlights the necessity for standardising nursing practices to ensure consistent and equitable empowering support for family members across different healthcare settings.
- Supports the critical role of nurses in facilitating family member empowerment through collaborative and interprofessional practices.
- Demonstrates practical implications for improving clinical practice and education by integrating family members' perspectives into care strategies.

## 1 Introduction

Traumatic brain injury (TBI) is a global health issue with significant implications and long‐term consequences for affected individuals, their families and healthcare systems (Maas et al. 2022). TBI is defined as a mild, moderate or severe disruption of brain function or structural damage caused by external physical forces, leading to a wide range of physical, cognitive, emotional and behavioural impairments (Khellaf, Khan, and Helmy 2019; Shahim and Zetterberg 2022). Moderate‐to‐severe TBIs typically require immediate hospitalisation during the acute phase (Manskow et al. 2018).

After hospital discharge, family members (FMs) often become the primary caregivers for patients with TBI, creating a complex and emotionally challenging experience requiring substantial adjustments in their roles and responsibilities (Bivona et al. 2020). FMs can feel powerless, uncertain, anxious and stressed in the acute phase (Lindlöf et al. 2023); thus, healthcare professionals are pivotal in supporting FMs of patients with TBI and promoting their coping during acute care (Bivona et al. 2020). Children especially struggle to understand post‐TBI symptoms, making it crucial that they receive more attention and support from healthcare professionals (Dawes et al. 2022). However, current studies indicate a lack in the implementation of empowering support (De Goumoëns et al. 2019; Lindlöf et al. 2023), partly due to inadequate nursing practice guidelines (Choustikova et al. 2020). In addition to needing adequate support and information about TBI and its consequences for the entire family's life, more attention must be paid to the discharge phase, which is currently perceived as deficient by FMs despite being one of the most crucial phases for FMs to cope at home with a person with a TBI person (Manskow et al. 2018). Thus, developing and standardising nursing practices that consider the FMs' needs and support healthcare professionals in empowering FMs more effectively is essential.

## 2 Background

Empowerment has been defined as both a process and an outcome (Friend and Sieloff 2018; Halvorsen et al. 2020). The empowerment process can be further divided into internal and external factors that influence the successful empowerment of FMs. External factors include the support received from the hospital environment where a patient is treated and healthcare professionals. In turn, these factors affect the internal factors of FMs, such as motivation and coping, thereby contributing to their empowerment (Nurhaeni et al. 2018). External factors influence the outcome of empowerment when the FMs perceive their ability to control and impact their situation (Sakanashi and Fujita 2017). Achieving this requires sufficient support, knowledge and guidance from healthcare professionals during the acute care phase. Nevertheless, healthcare professionals should recognise that empowerment is not just about providing information but also includes strategies that promote FMs' awareness of their personal and social situation‐specific resources and access to them (Halvorsen et al. 2020).

In the acute phase, FMs' empowerment process includes a dialogical and supportive relationship between FMs and healthcare professionals (Sakanashi and Fujita 2017; Wåhlin 2017), where FMs are seen as an essential part of comprehensive care planning of patients with TBI and implementation throughout the entire acute hospitalisation period (Lindlöf et al. 2023). However, Halvorsen et al. (2020) identified challenges in the empowerment process, including inequality between FMs and healthcare professionals. Healthcare professionals' patronage and inequality can act as barriers to the empowerment of FMs, or a lack of awareness of these factors can lead to the failure of empowering FMs. Therefore, there is a need for healthcare professionals to recognise the effect of the approaches they apply to empower FMs, particularly considering factors such as the initial state of shock FMs experience, which may hinder trust‐building (Lindlöf et al. 2023). Empowerment should always be defined within each specific context, considering all involved, such as patients, FMs and healthcare professionals (Wåhlin 2017).

There are only a few studies addressing the support of FMs during the acute phase for patients with TBI experiencing care conflicts. For example, as Coco et al. (2014) highlighted, nurses evaluated their support for FMs during the hospitalisation of patients with TBI as nearly excellent, whereas FMs reported inadequate and poor support during acute care (Choustikova et al. 2020; Lindlöf et al. 2023). However, research suggests that the high level of competence among healthcare professionals is crucial for the realisation of empowering support for FMs (De Goumoëns et al. 2022), making it essential to identify the conflicting factors that influence the implementation of empowering support.

There is a gap in the knowledge from FMs and nurses' perspectives regarding empowering support of FMs and its implementation during the acute care hospitalisation of patients with TBI. Thus, it is important to observe that the acute phase of care for patient with TBI lacks nursing recommendations and structured care approaches to guide healthcare professionals to support FMs equitably, regardless of where the first treatment for patients with TBI is provided.

## 3 The Study

### 3.1 Aim

This study aimed to investigate the perceptions of FMs of patients with TBI and nurses on empowering support and its implementation during the acute care phase, specifically within the context of neurosurgical and neurological care in Finnish hospital settings. Our focus was to identify possible similarities and differences between the perceptions of FMs and nurses.

The research questions were as follows: (i) What is empowering support during the acute care phase from the perspective of FMs of patients with TBI and nurses? (ii) How is empowering support implemented during the acute care phase from the perspective of FMs of patients with TBI and nurses?

## 4 Methods

### 4.1 Design

The study's design was based on a participatory qualitative descriptive approach, where FMs and nurses actively collaborated to construct shared knowledge. This approach ensures that diverse perspectives are included, allows for data collection from a larger group and empowers participants, enhancing the validity and depth of the findings (Löhr, Weinhardt, and Sieber 2020). The data were collected utilising the World Cafe method, which provided a structured framework while promoting active engagement and insightful discussions. This method helped explore the various dimensions of empowerment for FMs during the acute care of patients with TBI (Löhr, Weinhardt, and Sieber 2020). This study followed the Consolidated Criteria for Reporting Qualitative Research (COREQ) guidelines (Appendix S1) (Tong, Sainsbury, and Craig 2007).

### 4.2 Participants

This study was part of a broader participatory development and research project aimed at creating an empowering support model for FMs of patients with TBI. FMs and nurses were engaged in the project's design. FMs were recruited for the study through the project's social media channels, website and email. An email was sent to approximately 2300 Finnish Brain Injury Association members to reach potential participants, recognising that not all FMs are registered members. Initially, 12 volunteer FMs from across Finland expressed interest, with seven of whom eventually participated in the study. Among the participating FMs, diversity was observed in their relationship to the individual with TBI, with one being a sibling, one a parent, one a partner and four spouses. Their close person's TBI accident had occurred from 2 to 10 years prior. Regarding nurse participants, the project coordinator informed the hospital's head nurses of the neurosurgery and neurology acute care departments at four hospitals in the University Hospital district about the study and emailed the invitation letters to the nurses. 11 nurses were recruited from the ICU (*n* = 3), neurosurgical ward (*n* = 2) and neurological ward (*n* = 6), all women with more than 5 years of work experience as nurses in specialised health care (Table 1). Before the study began, FMs and nurses attended separate informational sessions where the study procedures were discussed in detail, both verbally and in written form. In addition, participants were informed that they could withdraw from the study at any time. Study participants' participation was based on their informed consent.

**TABLE 1:** Characteristics of participants (*N* = 18).

Characteristics	Total (*N* = 18)
**Family members**	***n* = 7**
Age	Range: 30–65 years
Gender	All female
Relationship to individual with TBI	
Sibling	1
Parent	1
Partner	1
Spouses	4
Time since injury	Range: 2–10 years ago
**Nurses**	***n* = 11**
Age	Range: 30–55 years
Gender	All females
Work experience	Over 5 years
University Hospital, Work settings	Hospitals (*N* = 4), Departments (*n* = 5)
Hospital A, ICU	3
Hospital A, neurosurgical ward	2
Hospital B, neurological ward	2
Hospital C, neurological ward	2
Hospital D, neurological ward	2

Abbreviations: ICU, intensive care unit; TBI, traumatic brain injury.

### 4.3 Data Collection

Separate workshops for data collection for both FMs and nurses were organised in November 2019 at the University of Applied Sciences facilities, which was a project partner. The workshops followed the World Café approach, facilitated by two neuro‐nursing lecturers from the University of Applied Sciences and the researcher responsible for data collection (JL). At the beginning of the workshop, JL explained the proceedings and the methods used for data collection, such as recording. Participants were divided into groups, with FMs split into two groups and nurses into three groups, engaging in discussions at three designated tables to discuss diverse perspectives on empowerment from the perspective of the bio‐physiological, functional, experiential, ethical, social and financial domains (Leino‐Kilpi et al. 2005) and their meaning in the context of acute care for patients with TBI. Each group appointed a chairperson who documented the discussions. The chairperson remained at the same table throughout the sessions and briefly summarised the previous group's discussion to the incoming participants, enriching and saturating the data. Each discussion lasted approximately 45 min before participants rotated to the next table, ensuring everyone engaged in every topic. The discussions lasted a total of 2 h and 15 min for each group. Following the table discussions, JL synthesised the notes and presented the findings to the participants, ensuring that their perspectives were accurately understood and that the chairpersons were informed about the discussions and outcomes of other tables (Löhr, Weinhardt, and Sieber 2020).

JL recorded the discussions at all the tables and transcribed them verbatim. Each participant was coded using a combination of letters and numbers, for example, FMs as FM1 and nurses as N1, facilitating the ability to track individual participants' comments during the analysis phase.

### 4.4 Data Analysis

**FIGURE 1:**
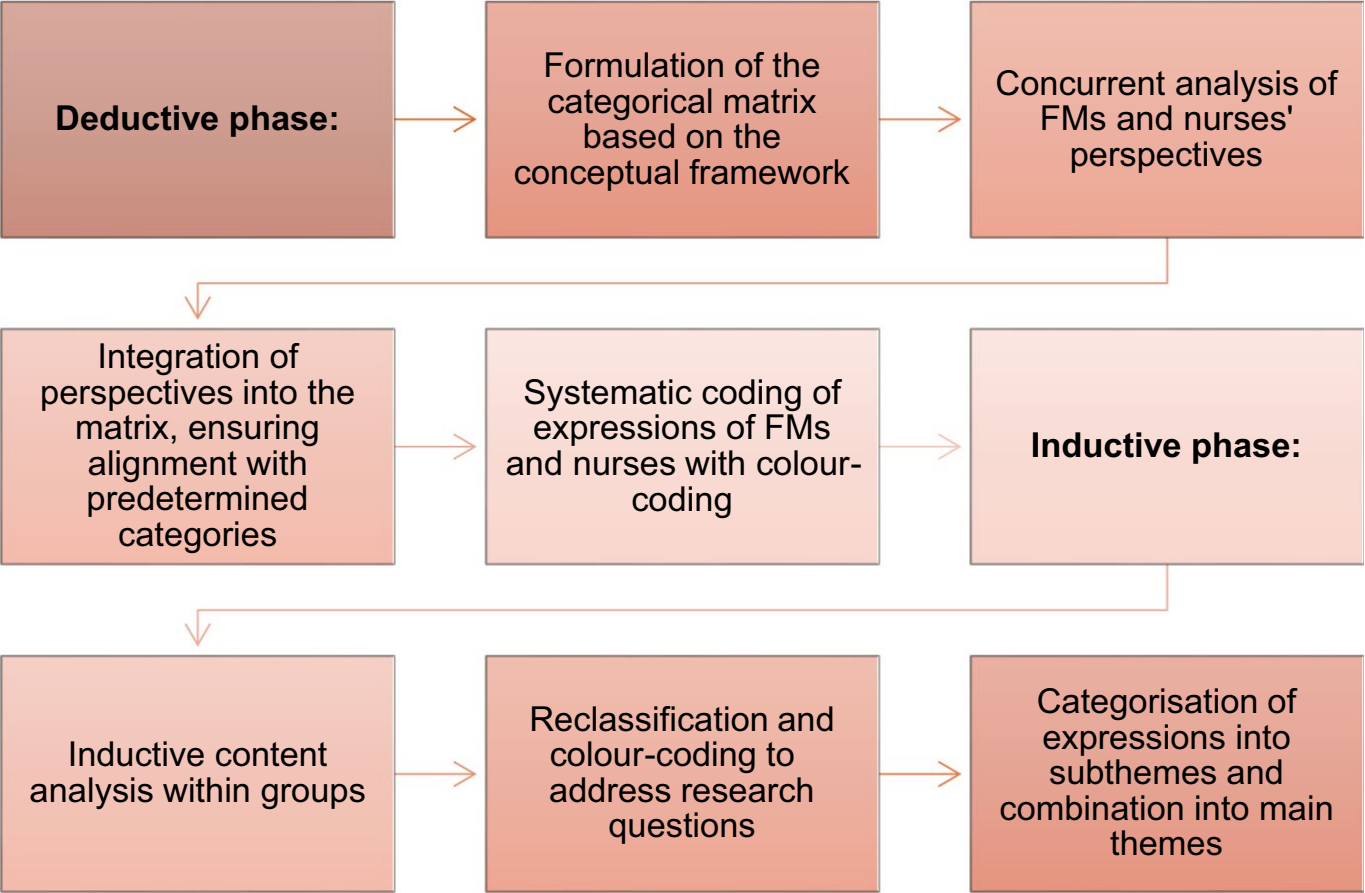
Abductive analysis process from deductive to inductive phase.

Employing an abductive approach, we sought to gain a deep understanding of the FMs’ and nurses' perceptions and to better understand their experiences regarding empowering support and its implementation in acute care. This approach allowed for flexible data exploration, enabling new insights and patterns that contribute to a rich understanding of the dynamics of empowering support during the acute care of patients with TBI (Timmermans and Tavory 2012). The abductive analyses proceeded from the deductive to the inductive phase. A description of the abductive analysis process from deductive to inductive phase is shown in (Figure 1).

The deductive analyses began with utilising a conceptual framework developed in a previous study regarding the empowering support for FMs of patients with TBI during the acute phase of hospital care (Lindlöf et al. 2023). This framework categorised the empowerment process of FMs into four themes regarding support: (a) needs‐based informational, (b) participatory, (c) competent and interprofessional, and (d) community support. The context encompasses FMs' experiences during the acute care phase of patients with TBI, including emergency care, intensive care unit (ICU) care and inpatient care.

The conceptual framework served as the base for developing the categorical matrix. First, JL initiated the analysis by thoroughly reading two transcribed texts from FMs and the nurses to gain familiarity with the content and to create an extensive overview of the data. Following this, both the perspectives of FMs and nurses were concurrently analysed, and expressions detailing empowerment narratives and sentences were transferred to the classification matrix using Excel. This facilitated the integration of their perspectives into the matrix, ensuring alignment with the predetermined categories within the conceptual framework. FMs and nurses' expressions were then systematically coded, with an additional step of colour‐coding implemented in Excel to differentiate between the viewpoints of FMs and nurses (Appendix S2).

In the second phase of inductive analysis, JL read the transferred text within the matrix to gain a deep understanding of the content. We selected units of analysis as sentences or parts of sentences and reduced the original expressions. The analysis continued within the groups using inductive content analysis. Subsequently, the reduced phrases were reclassified and colour‐coded to address the research questions. Expressions describing empowerment and its implementation were categorised into subthemes and combined into overarching themes representing FMs' empowerment and its implementation.

**FIGURE 2:**
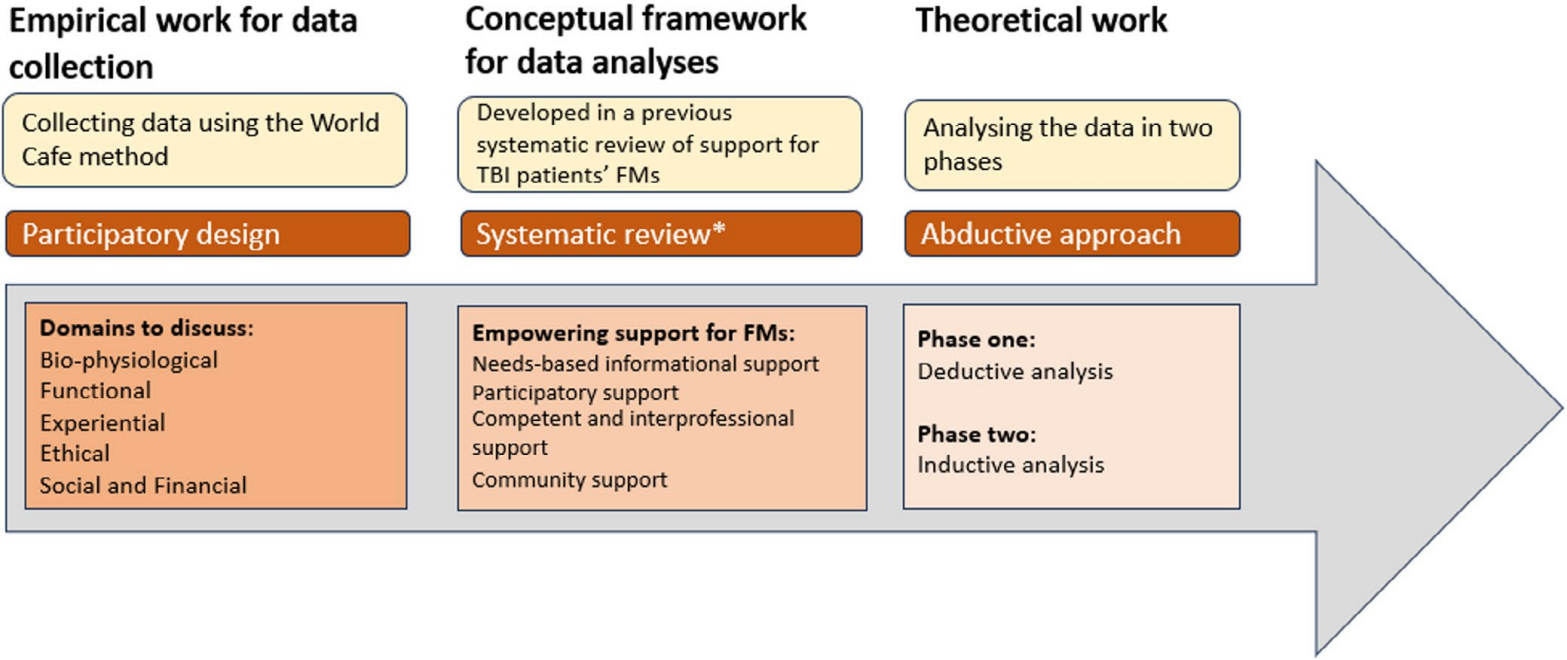
Process from participatory data collection to abductive analysis. *Lindlöf et al. (2023).

The process from participatory data collection to abductive analysis continued until saturation was reached on the prominent themes, illustrated in (Figure 2).

### 4.5 Ethical Considerations

University Hospital (HUS/163/28 August 2019) and the National Traumatic Brain Injury Association granted a research permit in September 2019. The participants were informed orally and in writing about the voluntary nature of their participation in the study, and the assurance of their anonymity. Additionally, they were informed of their right to withdraw from the study at any time.

### 4.6 Rigour

This study's rigour was maintained by following criteria (credibility, transferability and dependability and confirmability) all research phases (Johnson, Adkins, and Chauvin 2020). During the analysis phase, JL had primary responsibility, whereas HT, KC and TV provided critical assessments to enhance credibility. To improve the dependability and trustworthiness of the analysis, three researchers (JL, KC and JH) had substantial experience in the acute care of brain‐injured patients and interactions with their FMs. This experience provided the researchers with a deep understanding of the research context and participants, potentially enabling the identification of hidden meanings in participants' narratives. However, during the analysis process, the researchers tried to eliminate any influence of their nursing backgrounds on interpreting participants' reports. This helped to avoid bias in the analysis of results. Nevertheless, the research team members had diverse backgrounds, comprehensive experience in thematic analysis and familiarity with the studied phenomenon. Member checking was used to ensure the credibility and confirmability of the findings through ongoing discussions with participants to validate their perspectives (Erdmann and Potthoff 2023). The transferability of the results was addressed through detailed descriptions of the study participants and context, allowing for the evaluation of the usability of the results in other similar settings. The study does not aim for generalisability but provides valuable insights for similar healthcare environments.

Throughout the study, frequent meetings dedicated to examining and refining the identified themes from the data, as well as continuous and rigorous discussions, were held among all researchers (JL, HT, KC, TV, JH and SV). We maintained detailed documentation for all research phases.

## 5 Findings

Based on the abductive analysis, we identified four main themes describing empowering support of FMs and its implantation in the acute‐phase hospitalisation of patients with TBI: (1) FMs' diverse information and guidance needs of TBI, treatment and its impact on family life, (2) support based on empowering FMs in participation, competence and decision‐making, (3) empowering FMs through collaborative nursing practices and interprofessional support, and finally (4) internal and external hospital support enhancing and promoting the empowerment of FMs (Table 2). Additionally, (Table 3) presents the perceptions of FMs and nurses regarding empowering support and its implementation.

**TABLE 2:** Empowering support for family members (FMs) of patients with traumatic brain injury (TBI) and its implementation in acute care hospitals.

	Main themes	Elements of empowering support in acute care	Elements of empowering support implementation in acute care
Empowering support for FMs of patients with TBI and its implementation in acute care hospitals	5.1 FMs' diverse information and guidance needs of TBI, treatment and its impact on family life	5.1.1 Promoting primary information and first encounter for FMs while maintaining hope	Quick information of the accident Sustaining and preserving hope throughout the hospitalisation Involving children and adolescents in information sharing
		5.1.2 Ensuring clarity of information and regular updates on the patient's condition through interactions	In written and spoken form Engaging in conversations Guiding to reliable information
		5.1.3 Promoting understanding of TBI symptoms and their impact on the family's future	Information about TBI symptoms and consequences Information on the invisible symptoms of TBI
		5.1.4 Guiding, encouraging and promoting open communication among FMs and others	Guiding how to communicate about TBI to others Acknowledging children's crisis Encouraging open discussions about TBI Considering the family's current life situation
	5.2 Support based on empowering FMs in participation, competence and decision‐making	5.2.1 Involving FMs in the care process with definite guidelines for patients with TBI	Involving and considering the whole family Involving FMs with definite guidance Encouraging participation
		5.2.2 Ensuring uninterrupted information flow and continuity of care in patient transfers	Engaging in decision‐making Informing about patient transfers Preparing for the future Engaging in discussions
	5.3 Empowering FMs through collaborative nursing practices and interprofessional support	5.3.1 Promoting systematisation and automation of nursing practices to enhanced support for FMs	Utilising the interprofessional Collaboration Systematise nursing practices Organising care meetings
		5.3.2 Implementing ethical support for FMs' rights and responsibilities	Information on the FMs rights and responsibilities Utilising the interprofessional collaboration for managing practical matters
	5.4 Internal and external hospital support enhancing and promoting the empowerment of FMs	5.4.1 Ensuring the interactional relationship between FMs and nurses	Opportunity to share emotions with the nurse One‐on‐one discussions with nurses Interest in the well‐being of FMs Supporting FMs' well‐being with definite guidance
		5.4.2 Good support network as a confirming and complementary factor of FMs support	Mapping the support network of FMs Encouraging to seek help and support from friends
		5.4.3 Promoting guidance of FMs to support services and peer support	Automatic referral to support services Contacting support services together with the nurse Timely referral to peer support

**TABLE 3:** Family members' and nurses' perceptions on empowering support and its implementations.

Main themes	Family members' perceptions	Nurses' perceptions
5.1 FMs' diverse information and guidance needs of TBI, treatment and its impact on family life	Experiences with the timeliness of contact and information about the accident and diagnosis vary	The importance of quick information is emphasised, but practical implementation variations are noted
	Experiences with the timeliness of contact and information about the accident and diagnosis vary	The importance of quick information is emphasised, but practical implementation variations are noted
	The importance of involving the entire family to prevent misunderstandings or incomplete information is stressed	It is not explicitly addressed
	Maintaining hope is believed to be crucial for empowerment and is generally done successfully	There is agreement that maintaining hope is important, and it is generally felt to be successfully practised
	More detailed, comprehensive information about injuries, both verbally and in writing, is desired	The importance is recognised, but written materials are not always available; it is believed that detailed information is the responsibility of physicians
	The need for honest, reliable, regular and clear information is emphasised	There is agreement on importance; providing clear information is generally felt to be successful but the challenge because of the complexity of brain injuries is noted
	The importance of nurses staying to discuss information after doctor meetings is highlighted, but it is often found unfulfilled	It is felt that the shock state of family members hinders their ability to grasp information during the ICU stay
	There is a desire for more guidance to access reliable information	Family members are often directed to reliable sources, but guidance is not systematic and not provided to every family member
	More information about brain injury symptoms, particularly invisible symptoms, is desired	There is hesitation to predict the impact of brain injuries on patients and families due to uncertainty about recovery trajectories
	Clear and comprehensive information about TBI and its effects is wanted	Avoidance of saying too much or making promises to prevent misunderstandings and unrealistic expectations is common
	There is a highlighted need for guidance on communicating brain injury diagnosis to children and supporting them during crises	It is not explicitly addressed
	Professional encouragement to discuss the diagnosis openly is hoped for	It is not explicitly addressed
	Comprehensive consideration of family situations and guidance for supporting children outside the home (e.g., kindergartens, schools) is desired	It is not explicitly addressed
5.2 Support based on empowering FMs in participation, competence and decision‐making	Involving family members is seen as promoting their significance and empowerment, and increasing their expertise in patient care, facilitating home management	Concern about the well‐being of family members is expressed, as they manage both patient care and their everyday activities
	Nursing practices are felt to be patient‐centred, excluding family members from the care process	Family members are emphasised that patient care is not their responsibility, but willingness to participate is assessed
	The need for reviewing treatment events during ward transfers is highlighted	It is not explicitly addressed
	Comprehensive information about the new care facility is desired	It is believed that comprehensive information is provided, but gaps in knowing other care units' procedures are acknowledged
	Healthcare professionals are felt not to adequately care for family members' well‐being during transitions	Efforts are discussed to assess family members' ability to manage at home and provide options, emphasising that patients are not discharged unless family members are confident
5.3 Empowering FMs through collaborative nursing practices and interprofessional support	The importance of receiving comprehensive information and meeting different experts is highlighted	The need for systematic interprofessional collaboration to support family members is emphasised
	There is hope for the systematic integration of interprofessional care meetings into TBI patient care	It is believed that interprofessional care meetings could resolve unclear situations and answer family members' questions
	Family members feel they do not receive sufficient information about their rights and obligations, particularly when the patient's legal capacity is impaired	Nurses primarily discuss rights and responsibilities from the patient's perspective rather than from the family members' perspective
	The importance of social workers in facilitating access to information and support is stressed	The social worker's role is perceived as complementing the provision of information and support to family members
	Bureaucratic issues, such as patient data protection, are viewed as hindrances to obtaining empowering information	Data protection law is perceived as both facilitating and hindering information provision to family members
	The importance of one‐on‐one conversations is emphasised, though they are not always feasible	Challenges in offering one‐on‐one discussions due to time constraints are reported
5.4 Internal and external hospital support enhancing and promoting the empowerment of FMs	Desire for a coordinator or single point of contact in hospital	It is not explicitly addressed
	The significance of friends and a strong support network as empowering factors is emphasised	The importance of a robust support network for family members' well‐being and empowerment is recognised
	The need for a systematic approach to guide family members towards support services, such as counselling, is expressed, and automatic provision of support from the hospital is desired	It is believed that self‐initiation is more common in modern times, and providing materials from digital healthcare resources would suffice for family members to discover support services tailored to their needs
	Family members wish for the first contact with support services to occur together with a nurse or at the nurse's initiative, rather than being solely referred to the services	It is not explicitly addressed
	Peer support is viewed as significant in enhancing empowerment, and automatic information regarding third‐sector activities and peer support is desired	Varied experiences regarding referring family members to third‐sector activities and peer support are emphasised, often dependent on nurses' knowledge and experience

Abbreviations: FMs, family members; TBI, traumatic brain injury.

### 5.1 FMs' Diverse Information and Guidance Needs of TBI, Treatment and Its Impact on Family Life

The discussions revealed the necessity of providing timely and clear information, maintaining hope and understanding the implications of TBI on both the patient's and the family's future. Additionally, the discussion between FMs emphasised the importance of guiding open communication.

#### 5.1.1 Promoting Primary Information and First Encounter for FMs While Maintaining Hope

One factor influencing the empowering first encounter and primary information was the quick contact and information of FMs about the accident as well as information about the diagnosis of the patient's TBI. FMs' experiences of the primary information and first encounter varied. Nurses also emphasised the significance of quickly informing FMs, but they perceived variations in its practical implementation. One nurse recalled a situation from her workplace.

If no one has explained the diagnosis and the treatments we provide in the early stages, it is a really difficult situation. We have family members who have been in the hospital for five days, and they haven't even talked to the doctor once. (N11)

Both FMs and nurses believed that maintaining FMs' hope throughout the patient's hospitalisation was crucial in sustaining their empowerment. This was attributed to the inherent challenge of providing predictable patient recovery information, especially during ICU treatment. However, most FMs and nurses found that hope was maintained successfully in practice.

Family members also stressed the importance of involving the entire family in providing primary information. They feared that relying on just one person to understand the information might lead to misunderstandings, incomplete information or even distorted information as it was passed from one FM to another.

#### 5.1.2 Ensuring Clarity of Information and Regular Updates on the Patient's Condition Through Interactions

Family members hoped to receive more detailed and comprehensive information about the injuries from healthcare professionals, both verbally and in writing. Nurses also recognised the importance of providing written materials about brain injuries to FMs but noted that such resources were not always available in healthcare units. Nurses felt that providing detailed and comprehensive information about brain injuries was the physicians' responsibility. However, they believed they provided sufficient information about the patient's condition, current symptoms the brain injury caused and the procedures performed. Nevertheless, providing predictable information about the patient's recovery and symptoms was challenging because of the complexity of brain injuries.

Family members and nurses emphasised the importance of honest, reliable, current, regular and clear information about the patient's condition. However, FMs reported that information about brain injuries was not always presented in sufficiently clear language, which could lead to misunderstandings. Nurses agreed that clear language should always be used, and they felt generally successful in doing so. To avoid misunderstandings, FMs highlighted the importance of nurses staying to discuss the information with FMs after meeting with the doctor, but they often found this to be unfulfilled. Similarly, nurses felt that discussing and reviewing information about brain injuries occurred frequently, but they believed that FMs' shock state hindered the reception and internalisation of information, especially during the ICU stay.

The family wants that information, but they can't grasp it in that state of shock. Sometimes, we've received feedback that we haven't told them, and then I start thinking, ‘We've all talked about it here’. It just passes them by. (N8)

Although FMs did not feel their mental state affected their understanding, they desired more guidance to access reliable information, given their immense thirst for knowledge during the acute‐phase treatment of patients with TBI. Most nurses often directed FMs to reliable information sources, but this guidance lacked systematicity and was not provided to every FM.

#### 5.1.3 Promoting Understanding of TBI Symptoms and Their Impact on the Family's Future

Family members desired more information about brain injury symptoms and their effects on the patients' and families' daily lives. This pertained particularly to potential invisible symptoms associated with brain injury, such as impaired decision‐making, behavioural challenges, memory issues and fatigue. Despite often being invisible, these symptoms significantly impacted the well‐being of individuals with brain injuries and their families, affecting social relationships, work life and daily activities.

I would have liked to receive more information already in the hospital about what brain injury actually is. (FM7)

However, nurses were hesitant to predict how brain injuries would affect patients' and their families' lives because of uncertainty about recovery trajectories. Additionally, to avoid unnecessary concern among FMs, nurses were also cautious about overdiagnosing brain injuries and their associated symptoms, given that the sequelae of brain injury symptoms were diverse and individualised.

There's also the issue of mild brain injuries, to prevent overdiagnosis. The role of the nurse is somewhat about what one dares to say to whom… it's quite tricky. (N7)

Nurses mentioned that FMs often latched on to their words, which led them to avoid saying and promising too much. Nurses believed it was important that all aspects of the patient's well‐being were not given equal importance (e.g., nasogastric tube dislodgement vs. decreased level of consciousness due to a brain injury). As a result, they restricted the information provided to FMs.

#### 5.1.4 Guiding, Encouraging and Promoting Open Communication Among FMs and Others

Supporting interaction between FMs, including children, was highlighted in family discussions, with nurses emphasising this less. FMs pointed out that they required more guidance and support from healthcare professionals regarding how to communicate the diagnosis of a brain injury to children and how to support children during their crisis. Differing perspectives and understandings of brain injuries often lead to strained relationships between children and parents.

Family members felt uncertain about how and to whom they should discuss a brain injury diagnosis. Participants in the family group discussions also expressed shame. FMs feared that someone outside the family would find out about the brain injury. Their most significant concern was how others would react and whether they could accept the family's new situation.

I remember that brain injuries have sometimes been used as an insult to someone. So, it can bring a sense of shame because you don't know. So, of course, it should come from the healthcare personnel, explaining what it's about. That probably hasn't been thought of that way, but it would be really good if there is someone who is familiarity with how to tell children and young people. (FM2)

Family members hoped for professional encouragement to discuss the diagnosis openly with both FMs and others. FMs with children and adolescents discussed that healthcare professionals should consider the family's situation more comprehensively. They desired guidance to support children and adolescents outside the home, including establishing contact with places such as kindergartens and schools.

### 5.2 Support Based on Empowering FMs in Participation, Competence and Decision‐Making

Participants shared their views on FMs' role in the care process of patients with TBI, highlighting the importance of empowerment through involving FMs and developing their competence. In addition, uninterrupted information flow played a crucial role in promoting FMs' well‐being.

#### 5.2.1 Involving FMs in the Care Process With Definite Guidelines for Patients With TBI

Involving FMs was seen in both groups as promoting their significance and empowerment. In addition, involving FMs increased their expertise in matters related to the patient's care, which, in turn, facilitated the family's ability to manage at home with the individuals with TBI. It was important for FMs that healthcare professionals involved the entire family in the patient's care, not just the FM who visited the patient most often because family dynamics might change over time, and the responsibility for the patient's affairs could unexpectedly shift to another FM. In both the FMs' and the nurses' group discussions, involving FMs in the patient's care was described as related to providing clear instructions for concrete guidance, and it was perceived as being well‐implemented in practice.

However, FMs desired more encouragement to participate in care of patients with TBI. They hoped to be seen more strongly as part of the patient's care process. In their discussions, FMs described that current nursing practices were focused strongly on a disease‐centred approach, in which the FMs did not seem to be included in any manner. During the discussions, nurses mentioned that they could not consistently implement the continuous involvement of FMs in a patient's care because of the small size of the patient rooms.

I have a feeling that when he was in the hospital, the organization was running at its own pace, and the family member did not belong to it in any way. At most, they would say hello and goodbye to me, and, ‘There's your husband’. (FM5)

In contrast, nurses expressed concern about the well‐being of FMs because they felt that, in addition to caring for the patient, FMs were also managing their everyday activities. Nurses often emphasised to FMs that taking care of the patient was not their responsibility but also believed they could assess whether FMs were willing to participate in the patient's care.

#### 5.2.2 Ensuring an Uninterrupted Information Flow and the Continuity of Care in Patient Transfers

Family members had varying experiences regarding the extent to which they were involved in decision‐making related to patient transfers and the continuity of care. Most nurses acknowledged that, when feasible from a nursing perspective, they often or always considered the FMs' opinions and wishes regarding matters concerning the patient with TBI.

Family members valued being informed well in advance about upcoming patient transfers, although this was only accomplished for some FMs. In turn, nurses underlined that patient transfers often happened with very short notice, so they could not promptly inform FMs. To promote an empowering experience for FMs, FMs highlighted that during ward transfers, it was essential to review the events of the treatment period together with professionals. However, they felt this was rarely realised in practice.

Maybe at the time when you move from one place to another, so first you're in the intensive care unit, and then you move elsewhere, would there be a kind of debriefing where you would go through what has actually happened during these two weeks or three weeks? (FM3)

Most nurses, again, believed they provided comprehensive information about the forthcoming care facility to FMs but confessed they did not always know other care units' operating procedures when the patient's follow‐up care took place in another hospital. Transfers may include ward transfers within the same hospital, transfers between different healthcare facilities or discharges from the hospital to another care setting. These transfers between care units or facilities can introduce challenges in communication and coordination of care, particularly regarding the continuity of information and follow‐up care plans for the patient. Nurses also recalled that information gaps among healthcare professionals sometimes resulted in not knowing what had been agreed upon regarding the patient's transfer and follow‐up care.

Many FMs believed that healthcare professionals were not taking enough care in their well‐being as they left the hospital, and they hoped for uninterrupted support at various stages of care. Nurses also discussed situations related to ensuring the patient's ongoing care, and they agreed that ensuring patient care at home was not always possible because of practical factors (e.g., available resources of the services and opening hours). However, nurses reported making several efforts to assess the FMs' opinions regarding their ability to manage at home alone with the TBI person. They gave them options, including alternative options for ongoing care, emphasised that patients were not typically discharged unless the FMs were confident in their ability to cope.

### 5.3 Empowering FMs Through Collaborative Nursing Practices and Interprofessional Support

Participants reflected on empowering FMs through collaborative nursing practices and interprofessional support, underscoring the need to systematise nursing practices and automate them to provide enhanced support for FMs. Both groups pointed out the significance of a social worker was pointed out in discussions among both groups, with their role seen as complementing the ethical support provided to FMs.

#### 5.3.1 Promoting Systematisation and Automation of Nursing Practices to Enhanced Support for FMs

Both FMs and nurses highlighted the significance of interprofessional collaboration in facilitating comprehensive information and support for FMs. It was important for FMs to receive information and to have the opportunity to meet different experts during the patient's hospital care. The lack of systematic procedures for utilising interprofessional teams in supporting FMs became apparent in the discussions with nurses. These conversations revealed that not all hospital wards had interprofessional teams, and even on the wards that did, these teams' functioning was often unknown or underutilised for family support.

Most FMs and nurses pointed out that interprofessional collaboration should automatically be part of the care process for patients with TBI and integral to empowering FMs. Several FMs and nurses hoped that interprofessional care meetings would be systematically integrated into the care and nursing practices for patients with TBI. Nurses believed many unclear situations could be resolved, and FMs' questions could be answered through interprofessional care meetings.

Somehow, when you think about how significant it is, there should already be a meeting in the hospital when you have a traumatic brain injury. There should be a joint meeting, social workers and therapists. (N1)

I believe that many issues could be resolved this way, and you could ask different professionals about different things. (N3)

Nurses emphasised that interprofessional teams usually consisted only of professionals caring for patients with TBI. The patients and their FMs are rarely included in these teams. Care meetings are often held when the patient's health condition becomes challenging, but they are not automatically scheduled for other situations. FMs have also shared the view that care meetings are only organised when they request them, raising questions about the potential benefits of a more proactive approach. The systematic organisation of care meetings varies according to the department. FMs described their powerlessness in seeking and understanding aspects of the care for patients with TBI and wished for the automatisation of interprofessional care discussions as part of their information gathering and family support.

#### 5.3.2 Implementing Ethical Support for FMs' Rights and Responsibilities

Family members believed they did not receive sufficient information about their rights and obligations regarding caring for and making decisions on behalf of the patients with TBI and the entire family, particularly when the legal capacity of patients was impaired. Most FMs felt that healthcare professionals did not understand their responsibilities for family care and managing everyday family matters outside the hospital.

In contrast, nurses contemplated that ethical conduct was integral to nursing, making it challenging to focus on specific areas related to ethical support. Nurses primarily discussed rights and responsibilities from the perspective of patients with TBI rather than that of the FMs. However, most nurses believed that ethical support included listening to FMs, considering their opinions and involving them in decision‐making, but they also highlighted considering the patient's preferences.

The importance of social workers in facilitating FMs' access to information and support was stressed in both the FMs' and the nurses' group discussions. When a social worker was not automatically involved in providing information to FMs, FMs experienced challenges obtaining the necessary documents required for legal and practical matters, such as care certificates and authorisations. FMs also emphasised it was burdensome to navigate different procedures for submitting documents to the appropriate authorities because they did not receive sufficient guidance and support from healthcare professionals.

I did all the applications and this authorization. We had to evaluate if this was within the insurance coverage. I applied for sick leaves myself, and my husband was on temporary layoff, so I took care of those issues. There is a lot of work for a family member in that shock situation. (FM2)

In addition, FMs viewed bureaucratic issues, such as patient data protection, as a hindrance to obtaining empowering information. This was especially relevant when the FM was not a close relative of the patients with TBI (e.g., a partner). In the discussions among nurses, the data protection law was seen as both facilitating and hindering information provision to FMs. In complex family situations, the data protection law was perceived as facilitating the healthcare professionals' work because nurses could always refer to the regulations in information sharing. However, nurses emphasised they always tried to clarify unclear situations in cooperation with the FMs.

### 5.4 Internal and External Hospital Support Enhancing and Promoting the Empowerment of FMs

The discussions emphasised the importance of fostering a strong interactional relationship between FMs and nurses. Additionally, a good support network emerged as a confirming and complementary factor in supporting FMs, enabling them to navigate the challenges of caring for patients with TBI. Furthermore, participants identified the promotion of guidance for FMs to support services and peer support as crucial, with the issue of timely directing to these support services left at a deliberative level.

#### 5.4.1 Ensuring the Interactional Relationship Between FMs and Nurses

Both FMs and nurses identified factors contributing to FM empowerment during the hospitalisation of patients with TBI. These factors revolved around the FMs' ability to be heard, seen and accepted as they navigated their emotions throughout the hospitalisation of patients with TBI. Although the FMs' experiences in processing their emotions with healthcare professionals varied, FMs often hoped to receive more information about the emotional responses associated with the crisis phase and support in accepting those feelings. In contrast, nurses reported they almost always discussed potential emotional reactions with FMs and stressed that these feelings were entirely normal and acceptable.

Family members underlined the importance of automatically providing them with an opportunity for one‐on‐one conversations with healthcare professionals; however, in practice, this only occurred for some FMs. Nurses also recognised the significance of one‐on‐one discussions for helping FMs process their emotions but reported challenges in offering such discussions due to limited available time.

You have to do so many other things, so there's no time left for that meeting. That would help… because many issues could be resolved by talking with the patients and their families for ten minutes to calm and listen to them. Even if you don't necessarily have answers. But in some way, they feel heard. (N9)

Family members understood that healthcare professionals could not meet with them individually but hoped for a support system to be present at the hospital, especially during the ICU stay. In an ideal situation, FMs thought there should have been a coordinator or a single point of contact in the hospital whom they could approach at their convenience.

Family members and nurses held differing views on healthcare professionals' interest in FMs: well‐being and endurance. However, they both agreed that professionals were not sufficiently adept at recognising FMs' emotional support needs and providing them with the appropriate support services. Nurses stated that they did not always have enough knowledge and tools to support FMs according to their needs but acknowledged that their experiences and expertise played a significant role in their ability to assess their well‐being needs and support FMs. During their discussions, most nurses emphasised the importance of advising FMs to care for themselves. Nurses also encouraged FMs to gather their strength for the rehabilitation phase of patients with TBI because they believed that patient required more support from their loved ones during the rehabilitation process rather than in the acute phase of hospitalisation.

#### 5.4.2 Good Support Network as a Confirming and Complementary Factor of FMs' Support

The group discussions involving FMs emphasised the importance of friends and a good support network as factors empowering family resources. Particularly in situations where FMs felt they had received minimal emotional support from healthcare professionals, they highlighted friends' support. Similarly, in the group discussions with nurses, the significance of a strong support network for FMs' well‐being and empowerment was evident. Friends were seen as motivators, enabling FMs to take time away from caring for the patients with TBI and to gather strength for the future. Nurses observed a good support network complementing FMs' emotional support needs because they often felt that professional emotional support was lacking because of insufficient hospital resources. Nurses also mentioned that they aimed to assess the family's support network almost always and encouraged FMs to seek help from close relatives and friends for daily matters.

Some may have a very small support network. Others, on the other hand, may have a quite extensive one. It is beneficial to have many friends and close relationships; it gives strength to deal with those issues. (N6)

#### 5.4.3 Promoting Guidance of FMs to Support Services and Peer Support

The need for a systematic approach to guide FMs towards support services was a recurring theme in discussions involving FMs and nurses. FMs shared a common sentiment; they did not receive enough information about support services such as counselling from healthcare professionals and felt uncertain about how to access such services independently. FMs hoped that the initiative to provide support would come automatically from the hospital.

As family member you are the one that has to inquire about everything, and you have to know and be the one […] there should be individuals there to assist so that you don't have to Google everything but rather have someone for whom this is a job. (FM4)

In addition to or instead of referring FMs to support services, FMs wished for the first contact with these services to take place together with a nurse or at the nurse's initiative. Nurses, instead, believed that self‐initiation was more common in modern times. They thought that providing FMs with materials from digital healthcare resources would be sufficient for them to discover support services tailored to their needs. Nevertheless, nurses underlined that they did not have enough knowledge about support services to guide FMs more concretely and in a needs‐based manner.

Peer support was seen as a significant factor in enhancing FMs' empowerment in discussions with both FMs and nurses. FMs desired to receive information automatically regarding third‐sector activities and peer support, although there were uncertainties about the optimal timing for such support. The timing of referral to peer support was deliberated in discussions with both FMs and nurses. Experiences among nurses varied regarding referring FMs to third‐sector activities and peer support, often dependent on their knowledge and experience. Additionally, nurses believed that third‐sector services might not be necessary if the patients with TBI showed positive recovery progress, and if required, FMs would find them independently. In contrast, FMs highlighted their ability to assess their need for peer support and third‐sector services if they had received sufficient information from healthcare professionals.

## 6 Discussion

We explored the perspectives of FMs and nurses on empowering support and their experiences implementing it in the acute‐phase care of patients with TBI in the hospital using participatory methods. Our study revealed a general agreement among FMs and nurses on the components of empowering support, including a holistic first encounter, primary information, systematic guidance, participation in the care process of patients with TBI, decision‐making and discharge, alongside interprofessional support. However, a discrepancy emerged in the FMs' and nurses' perspectives regarding the consideration and involvement of the entire family in the care for patients with TBI, negatively affecting the practical implementation of empowering support in nursing practice. These findings align with previous studies (Dawes et al. 2022; De Goumoëns et al. 2019; Lindlöf et al. 2023), suggesting a need for nursing education to focus on FMs' needs, promoting a more family‐centred approach (Dawes et al. 2022; Wetzig and Mitchell 2017).

Family members reported needs that are insufficiently met. Nurses, however, perceived their support for the families as relatively successful. The conflicting experiences between FMs and nurses may stem from the absence of standardised, yet patient‐ and family‐centred nursing practices, resource deficits, high turnover rates among nurses and internal communication challenges (Choustikova et al. 2020; Walker, Schlebusch, and Gaede 2021). Nurses explored these issues when reflecting on the understanding and implementation of empowering support. Our research reveals that the absence of uniform nursing practices, as the nurses discussed, has created inequalities among FMs regarding information and support implementation. Nurses' conversations revealed various experiences, such as the systematicity of contacting FMs and responsible parties. Yet, it is crucial to acknowledge that FMs often do not fully comprehend nurses' time constraints and other resource limitations (Walker, Schlebusch, and Gaede 2021), potentially explaining why this theme was not explicitly addressed in FMs' discussions. In contrast, FMs' differing perspectives regarding the information and support received presumably stem from the absence of systematic patient‐ and family‐centred nursing practices specifically (Lindlöf et al. 2023).

The lack of a clear definition of the roles of healthcare professionals in providing information has resulted in confusion among both FMs and nurses. This confusion mainly regards who is primarily responsible for conveying detailed information about TBIs, the patient's condition, treatment and the potential impacts of TBI symptoms on family life. Numerous studies have emphasised the imperative of clearer communication between FMs and professionals as a pivotal factor in empowering FMs (Hayes et al. 2023; Kreitzer et al. 2019; Walker, Schlebusch, and Gaede 2021). However, in our study, nurses largely perceived themselves as providing comprehensive and clear information about the current condition of patients with TBI. Unclear professional roles also emerged among members of multidisciplinary teams, despite both FMs and nurses acknowledging that a multidisciplinary team complemented the information and support needs of FMs, especially in hospitals employing multidisciplinary approaches (De Goumoëns et al. 2019).

According to our research findings, there is an emphasised need among FMs for dedicated support in the hospital, ensuring sufficient information and guidance throughout the care of patients with TBI. This need likely arose from the previously mentioned ambiguous professional roles and inadequate communication. These results align with several earlier studies (Holloway, Orr, and Clark‐Wilson 2019; Lindlöf et al. 2023; Walker, Schlebusch, and Gaede 2021). Efforts to address these issues have been pursued by developing diverse acute‐phase family interventions (De Goumoëns et al. 2022; Naef, Massarotto, and Petry 2020), employing a nurse‐led approach to support families. Intervention outcomes demonstrated that advanced family nursing practices facilitated meeting the needs for support and information among FMs while simultaneously easing the workload for nurses during acute care. We believe having a family nurse can benefit both FMs and nurses. However, more research is required to ensure that such family nurses and interventions also effectively cater to the needs of children in families and encompass all aspects of empowering support.

Both FMs and nurses express the need for up‐to‐date and regular information assurance through collaborative care meetings involving professionals responsible for patients with TBI, including healthcare professionals and FMs. Presently, these care meetings occur sporadically or require FMs' specific requests. FMs believe such meetings could facilitate their adaptation to the ward transfers of patients with TBI and improve discharge situations. These aspects are deemed crucial for the empowerment of FMs and their perceived ability to manage individual with TBI at home (De Goumoëns et al. 2019; Lutz et al. 2022). Prior studies have noted FMs' involvement in the treatment process of patients with TBI and decision‐making, particularly concerning continuous care plans (Hayes et al. 2023; Manskow et al. 2018). To address this issue, it is important to recognise that the responsibility for conveying information should not rest solely on one professional group. Instead, it should be a collaborative effort among all multidisciplinary team members (Wade, Nayar, and Haider 2022). As highlighted in our study, each team member, including doctors, nurses, therapists and social workers, plays a critical role in ensuring that FMs receive consistent and comprehensive information. However, empowerment must always be studied within its specific context and among the participants involved in the research (Wåhlin 2017). Therefore, future studies are needed to gain more detailed insights into empowering support in the context of acute care for patients with TBI from the perspectives of various professional groups. This would help in understanding how the support for FMs can be integrated into the inter‐ and multidisciplinary team's activities.

Nonetheless, the practical implementation of this involvement encounters challenges because of difficulties in recognising FMs' rights and responsibilities, as identified in discussions with both FMs and nurses. FMs' perceptions of inadequate ethical support align closely with nurses' uncertain perspectives on providing ethical support. Identifying ethical support aspects was notably more straightforward for nurses when adopting the patient's viewpoint, reinforcing the prevailing patient‐centred nature of nursing practices (Walker, Schlebusch, and Gaede 2021). Nursing practices require updating and adopting new approaches to enhance family involvement in the patient's overall care (De Goumoëns et al. 2019). This entails a shift towards recognising and addressing FMs' needs and perspectives in the care process. Incorporating family‐centred care principles, education and training programmes for nurses could improve family engagement and empowerment, addressing the challenges identified in discussions with both FMs and nurses. This evolution in nursing practices could be vital for achieving a comprehensive and family‐centred approach (Lindlöf et al. 2023).

Ensuring the continuity of care for patients with TBI and guiding FMs towards essential support services were significant concerns discussed among both FMs and nurses. However, the realisation of this aspect frequently encountered challenges beyond nurses' control, such as bureaucratic hurdles and internal and external knowledge gaps within the hospital environment (Kreitzer et al. 2019; Lindlöf et al. 2023). Our findings have led to a novel insight that achieving support for FMs cannot be solely attributed to the interactions between FMs and nurses within the multi‐ or interdisciplinary context. It is rather intricately connected with internal and external factors within the hospital, over which nurses often have limited influence. These factors include the existing support network of FMs and the practices of external entities. Consequently, FMs' personal empowerment process during acute care is interconnected with organisational and community factors (Friend and Sieloff 2018). To promote the empowerment of FMs, collaboration across boundaries between the hospital and other entities should be strengthened. Early communication within the hospital and among various stakeholders is essential for achieving shared goals and fostering the empowerment of FMs (Eliacin et al. 2022).

Family members underscored the importance of guidance occurring automatically, without the need for them to inquire or independently seek available services and information. In contrast, nurses perceived contemporary practices as emphasising self‐direction, attributing this to the comprehensiveness of modern digital resources and materials. Despite the consensus in nurses' perspectives, variations were identified among nurses regarding whether they actively directed FMs to support services, such as peer support. Nurses' approaches to supporting and guiding FMs often relied on their individual work experience and competence, as evidenced in previous studies (Coco et al. 2014; Lindlöf et al. 2023). The identified disparities underscore the need for unified and evidence‐based nursing recommendations, ensuring consistent and effective support for FMs in acute care contexts.

### 6.1 Strengths and Limitations

This research demonstrates considerable strength as the first participatory study involving both FMs of patients with TBI and nurses, providing a comprehensive exploration of empowering support in nursing practice. This methodological choice provided an in‐depth understanding of the empowering support concept and its practical implementation. The participatory approach unveiled various factors influencing the successful execution of empowering support in nursing. However, some of the FMs who were initially interested in participating did not actually participate, which could be attributed to their limited resources to engage in the study, leading to participant cancellations. It is important to note that the recruitment of FMs through the project's social media channels, website and email may not represent purposeful sampling, potentially introducing bias by attracting only those who are active online or have the means to travel. This method could lead to a lack of diversity in the sample, as it may not capture FMs who are less connected to online platforms or have limited resources for participation. However, the data collected from FMs yielded substantial findings, and robust, firm and well‐founded results were achieved through concurrent data collection and analysis, highlighting significant consistencies in the gathered information. On the other hand, recruiting nurses primarily from specific hospitals with extensive experience in neurosurgical patient care and interaction with FMs may limit the applicability of the findings beyond these settings, potentially affecting their transferability and introducing bias in nurses' experiences with providing empowering support. It is worth noting that nurses with substantial work experience likely offered more profound insights into empowering support and a better understanding of factors influencing its implementation. Limitation to the transferability of FMs' perspectives due to the preponderance of female study participants was observed. Hence, the results cannot be applied to men and children. It remains unknown whether male FMs hold different perspectives compared with their female counterparts. Despite this constraint, efforts were made to enhance reliability by incorporating participants' original expressions into reporting results.

## 7 Conclusion

Our study, conducted through participatory research methods, underscores the lack of clear and standardised nursing practices across all themes, significantly influencing FMs and nurses' perspectives on empowering support and its practical implementation. Notably, disparities between FMs and nurses primarily concern the implementation of empowering support rather than its components. Although organisational and community factors heavily impact empowering support for FMs, the pivotal role of nurses in fostering family empowerment cannot be overstated. However, further research is needed to explore the significance of nurses' empowerment experiences and the influence of the nurse–organisation relationship on practical implementation. Our findings advocate for a shift towards family‐centred nursing practices, acknowledging the specific needs of children and the overall family situation. Importantly, our study emphasises the need for evidence‐based nursing recommendations to ensure equitable implementation of empowering support while recognising and valuing the competency and experience of individual nurses. However, it is imperative that such support is not solely dependent on individual nurse experiences or the care setting but rather guided by evidence‐based practices. Additionally, improved collaboration between organisations and different professional groups is highlighted as crucial for FMs' well‐being and resilience as well as overall family coping at home.

## Author Contributions

J.L., H.T., K.C. and T.V. made substantial contributions to the conception and design, or acquisition of data, or analysis and interpretation of data. J.L, H.T., K.C., J.H., S.V. and T.V. involved in drafting the manuscript or revising it critically for important intellectual content, given final approval of the version to be published, should have participated sufficiently in the work to take public responsibility for appropriate portions of the content and agreed to be accountable for all aspects of the work in ensuring that questions related to the accuracy or integrity of any part of the work are appropriately investigated and resolved.

## Conflicts of Interest

The authors declare no conflicts of interest.

## Supporting information


Appendix S1.

Appendix S2.

## Data Availability

Data openly available in a public repository that issues datasets with DOIs.

## References

[jan16424-bib-0001] Bivona, U. , D. Villalobos , M. De Luca , et al. 2020. “Psychological Status and Role of Caregivers in the Neuro‐Rehabilitation of Patients With Severe Acquired Brain Injury (ABI).” Brain Injury 34, no. 13–14: 1714–1722. 33190555 10.1080/02699052.2020.1812002

[jan16424-bib-0002] Choustikova, J. , H. Turunen , H. Tuominen‐Salo , and K. Coco . 2020. “Traumatic Brain Injury patients' Family Members' Evaluations of the Social Support Provided by Healthcare Professionals in Acute Care Hospitals.” Journal of Clinical Nursing 29, no. 17–18: 3325–3335. 32497326 10.1111/jocn.15359

[jan16424-bib-0003] Coco, K. , K. Tossavainen , J. E. Jääskeläinen , and H. Turunen . 2014. “The Provision of Emotional Support to the Families of Traumatic Brain Injury Patients: Perspectives of Finnish Nurses.” Journal of Clinical Nursing 22, no. 9–10: 1467–1476. 10.1111/jocn.12136 23489840

[jan16424-bib-0004] Dawes, K. , A. Carlino , M. van den Berg , and M. Killington . 2022. “Life Altering Effects on Children When a Family Member Has an Acquired Brain Injury: A Qualitative Exploration of Child and Family Perceptions.” Disability and Rehabilitation 44, no. 2: 282–290. 32427005 10.1080/09638288.2020.1766582

[jan16424-bib-0005] De Goumoëns, V. , K. Ayigah , D. Joye , P. Ryvlin , and A. S. Ramelet . 2022. “The Development of an Early Intervention for Supporting Families of Persons With Acquired Brain Injuries: The SAFIR Intervention.” Journal of Family Nursing 28, no. 1: 6–16. 34617490 10.1177/10748407211048217 PMC8814967

[jan16424-bib-0006] De Goumoëns, V. , A. Didier , C. Mabire , M. Shaha , and K. Diserens . 2019. “Families' Needs of Patients With Acquired Brain Injury: Acute Phase and Rehabilitation.” Rehabilitation Nursing Journal 44, no. 6: 319–327. 10.1097/rnj.0000000000000122 29300227

[jan16424-bib-0007] Eliacin, J. , S. K. Fortney , N. A. Rattray , and J. Kean . 2022. “Patients' and Caregivers' Perspectives on Healthcare Navigation in Central Indiana, USA After Brain Injury.” Health & Social Care in the Community 30, no. 3: 988–997. 33471969 10.1111/hsc.13275 PMC12063566

[jan16424-bib-0008] Erdmann, A. , and S. Potthoff . 2023. “Decision Criteria for the Ethically Reflected Choice of a Member Check Method in Qualitative Research: A Proposal for Discussion.” International Journal of Qualitative Methods 22: 7664. 10.1177/16094069231177664.

[jan16424-bib-0009] Friend, M. L. , and C. L. Sieloff . 2018. “Empowerment in Nursing Literature: An Update and Look to the Future.” Nursing Science Quarterly 31, no. 4: 355–361. 30223743 10.1177/0894318418792887

[jan16424-bib-0010] Halvorsen, K. , A. Dihle , C. Hansen , et al. 2020. “Empowerment in Healthcare: A Thematic Synthesis and Critical Discussion of Concept Analyses of Empowerment.” Patient Education and Counseling 103, no. 7: 1263–1271. 32164960 10.1016/j.pec.2020.02.017

[jan16424-bib-0011] Hayes, K. , S. Harding , K. Buckley , B. Blackwood , and J. M. Latour . 2023. “Exploring the Experiences of Family Members When a Patient Is Admitted to the ICU With a Severe Traumatic Brain Injury: A Scoping Review.” Journal of Clinical Medicine 12, no. 13: 4197. 37445232 10.3390/jcm12134197 PMC10342526

[jan16424-bib-0012] Holloway, M. , D. Orr , and J. Clark‐Wilson . 2019. “Experiences of Challenges and Support Among Family Members of People With Acquired Brain Injury: A Qualitative Study in the UK.” Brain Injury 33, no. 4: 401–411. 30663417 10.1080/02699052.2019.1566967

[jan16424-bib-0013] Johnson, J. L. , D. Adkins , and S. Chauvin . 2020. “A Review of the Quality Indicators of Rigor in Qualitative Research.” American Journal of Pharmaceutical Education 84, no. 1: 7120. 32292186 10.5688/ajpe7120 PMC7055404

[jan16424-bib-0014] Khellaf, A. , D. Z. Khan , and A. Helmy . 2019. “Recent Advances in Traumatic Brain Injury.” Journal of Neurology 266: 2878–2889. 31563989 10.1007/s00415-019-09541-4 PMC6803592

[jan16424-bib-0015] Kreitzer, N. , T. Bakas , B. Kurowski , et al. 2019. “The Experience of Caregivers Following a Moderate to Severe Traumatic Brain Injury Requiring ICU Admission.” Journal of Head Trauma Rehabilitation 35, no. 3: E299–E309. 10.1097/HTR.0000000000000525. PMC10346118 31479080

[jan16424-bib-0016] Leino‐Kilpi, H. , K. M. R. N. Johansson , K. M. R. N. Heikkinen , A. Kaljonen , H. M. R. N. Virtanen , and S. Salanterä . 2005. “Patient Education and Health‐Related Quality of Life: Surgical Hospital Patients as a Case in Point.” Journal of Nursing Care Quality 20, no. 4: 307–316. 16177581 10.1097/00001786-200510000-00005

[jan16424-bib-0017] Lindlöf, J. , H. Turunen , T. Välimäki , J. Huhtakangas , S. Verhaeghe , and K. Coco . 2023. “Empowering Support for Family Members of Brain Injury Patients in the Acute Phase of Hospital Care: A Mixed‐Methods Systematic Review.” Journal of Family Nursing 30, no. 1: 50–67. 10.1177/10748407231171933. 37191257 PMC10788044

[jan16424-bib-0018] Löhr, K. , M. Weinhardt , and S. Sieber . 2020. “The “World Café” as a Participatory Method for Collecting Qualitative Data.” International Journal of Qualitative Methods 19, no. 1: 6976. 10.1177/1609406920916976.

[jan16424-bib-0019] Lutz, A. M. , K. M. Warehime , A. B. Woods , et al. 2022. “Implementation of Interprofessional Meetings Preparing Caregivers of Patients With Brain Injury for Discharge: A Pilot Study.” Professional Case Management 27, no. 5: 239–245. 35901256 10.1097/NCM.0000000000000562

[jan16424-bib-0020] Maas, A. I. , D. K. Menon , G. T. Manley , et al. 2022. “Traumatic Brain Injury: Progress and Challenges in Prevention, Clinical Care, and Research.” Lancet Neurology 21, no. 11: 1004–1060. 36183712 10.1016/S1474-4422(22)00309-X PMC10427240

[jan16424-bib-0021] Manskow, U. S. , C. Arntzen , E. Damsgård , et al. 2018. “Family Members' Experience With in‐Hospital Health Care After Severe Traumatic Brain Injury: A National Multicentre Study.” BMC Health Services Research 18, no. 1: 1–10. 30526574 10.1186/s12913-018-3773-7 PMC6286568

[jan16424-bib-0022] Naef, R. , P. Massarotto , and H. Petry . 2020. “Family and Health Professional Experience With a Nurse‐Led Family Support Intervention in ICU: A Qualitative Evaluation Study.” Intensive & Critical Care Nursing 61, no. 102: 916. 10.1016/j.iccn.2020.102916 32807604

[jan16424-bib-0023] Nurhaeni, N. , Y. Rustina , N. Agustini , and N. E. Rosuliana . 2018. “Impact of Family Empowerment Model on Satisfaction and Children's Length of Stay in Hospital.” Enfermería Clínica 28: 36–40.29153438

[jan16424-bib-0024] Sakanashi, S. , and K. Fujita . 2017. “Empowerment of Family Caregivers of Adults and Elderly Persons: A Concept Analysis.” International Journal of Nursing Practice 23, no. 5: e12573. 10.1111/ijn.12573 28691266

[jan16424-bib-0025] Shahim, P. , and H. Zetterberg . 2022. “Neurochemical Markers of Traumatic Brain Injury: Relevance to Acute Diagnostics, Disease Monitoring, and Neuropsychiatric Outcome Prediction.” Biological Psychiatry 91, no. 5: 405–412. 34857362 10.1016/j.biopsych.2021.10.010

[jan16424-bib-0026] Timmermans, S. , and I. Tavory . 2012. “Theory Construction in Qualitative Research: From Grounded Theory to Abductive Analysis.” Sociological Theory 30, no. 3: 167–186.

[jan16424-bib-0027] Tong, A. , P. Sainsbury , and J. Craig . 2007. “Consolidated Criteria for Reporting Qualitative Research (COREQ): A 32‐Item Checklist for Interviews and Focus Groups.” International Journal for Quality in Health Care 19, no. 6: 349–357. 17872937 10.1093/intqhc/mzm042

[jan16424-bib-0028] Wade, D. T. , M. Nayar , and J. Haider . 2022. “Management of Traumatic Brain Injury: Practical Development of a Recent Proposal.” Clinical Medicine 22, no. 4: 353–357. 35705451 10.7861/clinmed.2021-0719 PMC9345207

[jan16424-bib-0029] Wåhlin, I. 2017. “Empowerment in Critical Care –A Concept Analysis.” Scandinavian Journal of Caring Sciences 31, no. 1: 164–174. 27164009 10.1111/scs.12331

[jan16424-bib-0030] Walker, J. , L. Schlebusch , and B. Gaede . 2021. “Support for Family Members Who Are Caregivers to Relatives With Acquired Brain Injury.” Journal of Mind and Medical Sciences 8, no. 1: 76–85.

[jan16424-bib-0031] Wetzig, K. , and M. Mitchell . 2017. “The Needs of Families of ICU Trauma Patients: An Integrative Review.” Intensive & Critical Care Nursing 41: 63–70. 28366520 10.1016/j.iccn.2017.02.006

