# Supplementary material for: Empowering Support for Family Members of Patients With Traumatic Brain Injury During the Acute Care: Insights From Family Members and Nurses
Source: J Adv Nurs. 2024 Sep 5;81(11):7674–89. doi: 10.1111/jan.16424 (PMC12535323; doi:10.1111/jan.16424)
Supplement: Supplementary file 2 — Appendix S2. [file JAN-81-7674-s001.docx]

Supplementary File 2. Example of deductive-inductive content analysis.

| **TBI patients' family members empowering support in acute care hospitals** |
| --- |

| **Needs-based informational** | **Participatory** | **Competent and interprofessional** | **Community support** | **Unclassified theme** |
| --- | --- | --- | --- | --- |
| Promptly informing family members about the accident and traumatic brain injury diagnosis | Encouraging the entire family to participate in the patient's care from the early phases | Interprofessional collaboration facilitated the managing of practical matters for family members | Nurses did not always know where family members could be directed for support services | Providing information and support for practical and legal matters |
| Providing verbal and written information about traumatic brain injuries | Providing concrete instructions for family involvement in the patient's care | The social worker's influence on family members' adaptation and coping during and after hospitalization | Nurses ensured that family members had access to a strong support network and emphasized its importance for family members' well-being | Hospitals' practices differ in how family members are informed and supported |
| Providing real-time and honest updates on the patient's progress and condition | Nurses' ability to recognize the needs and willingness of family members to participate in the patient's care | Nurses' professional competence was reflected in assessing and providing emotional support for family members | Referral to peer support should be automatic during the hospitalization period | Guiding family members in obtaining sick leave and certificates/documents |
| Informing family members about cognitive symptoms resulting from brain injury | Preparing and acquainting family members with the new care unit | Nurses recognized the importance of family members feeling heard for their well-being | Support from friends and family members helped family members cope with a challenging life situation | The legal basis for the right to access information and its significance in nurses' information communication to family members |
| Emphasizing the importance of discussing the treatment process, even when precise details could not be provided | Involving family members in decision-making regarding the patient's further treatment | Effective communication promotes mutual understanding and empathetic interaction between family members and nurses | Guiding family members to reliable sources of information where they could independently seek support services and peer support information | The lack of support and the opportunity for family members to discuss and share their feelings with nurses |
